# Supplementary material for: Genome-Wide Association Study Revealed SNP Alleles Associated with Seed Size Traits in African Yam Bean (Sphenostylis stenocarpa (Hochst ex. A. Rich.) Harms)
Source: Genes (Basel). 2022 Dec 13;13(12):2350. doi: 10.3390/genes13122350 (PMC9777823; doi:10.3390/genes13122350)
Supplement: Supplementary file 1 [file genes-13-02350-s001.zip › Olomitutu et al. Supplementary Figures.pdf]

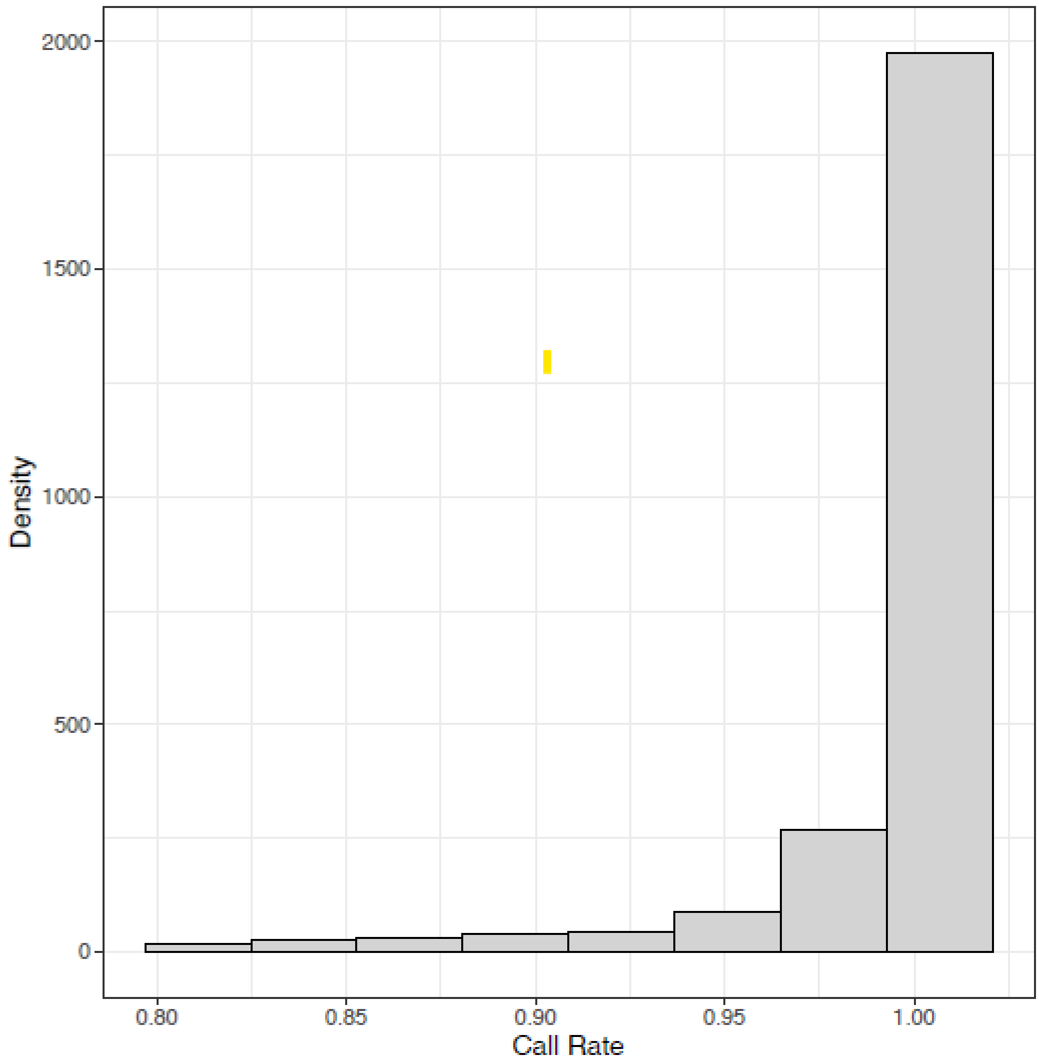

Supplementary Figure S1. Call rate distribution in the AYB Population

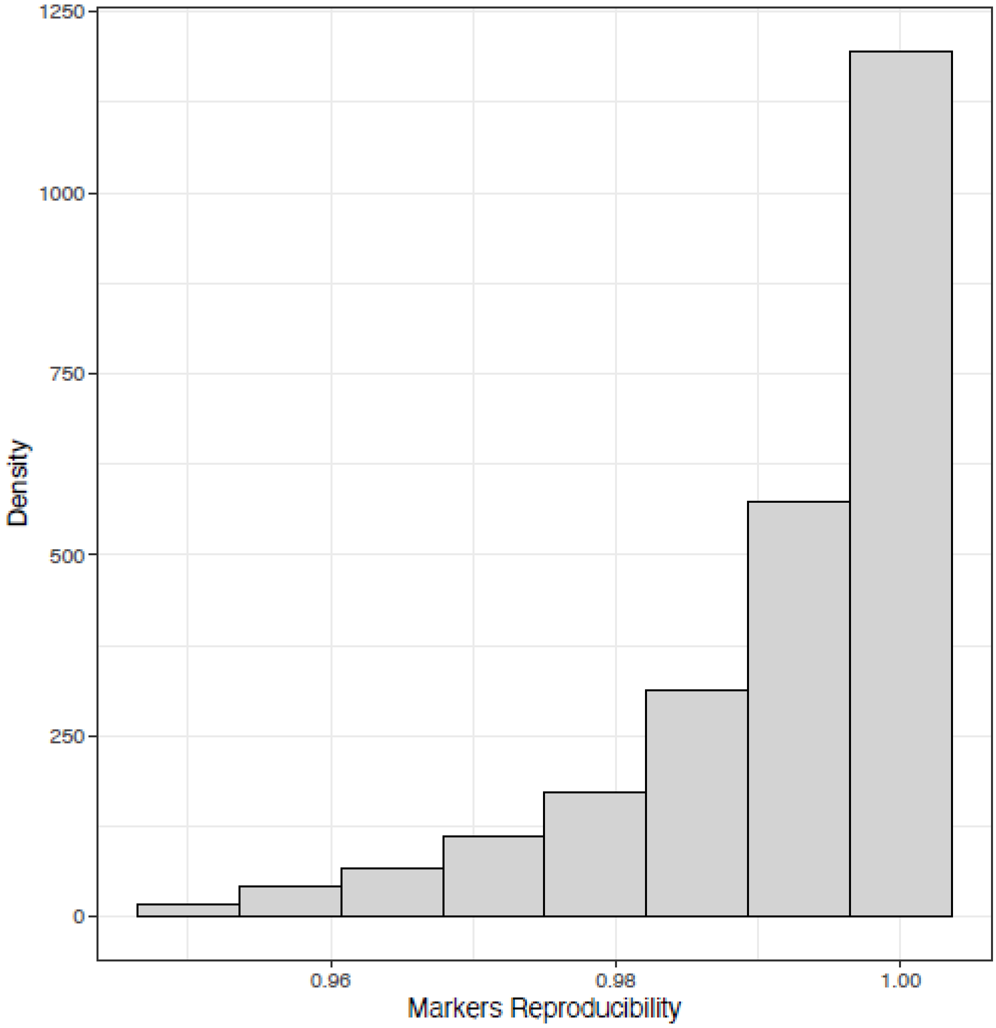

Supplementary Figure S2. Marker reproducibility distribution in the AYB population

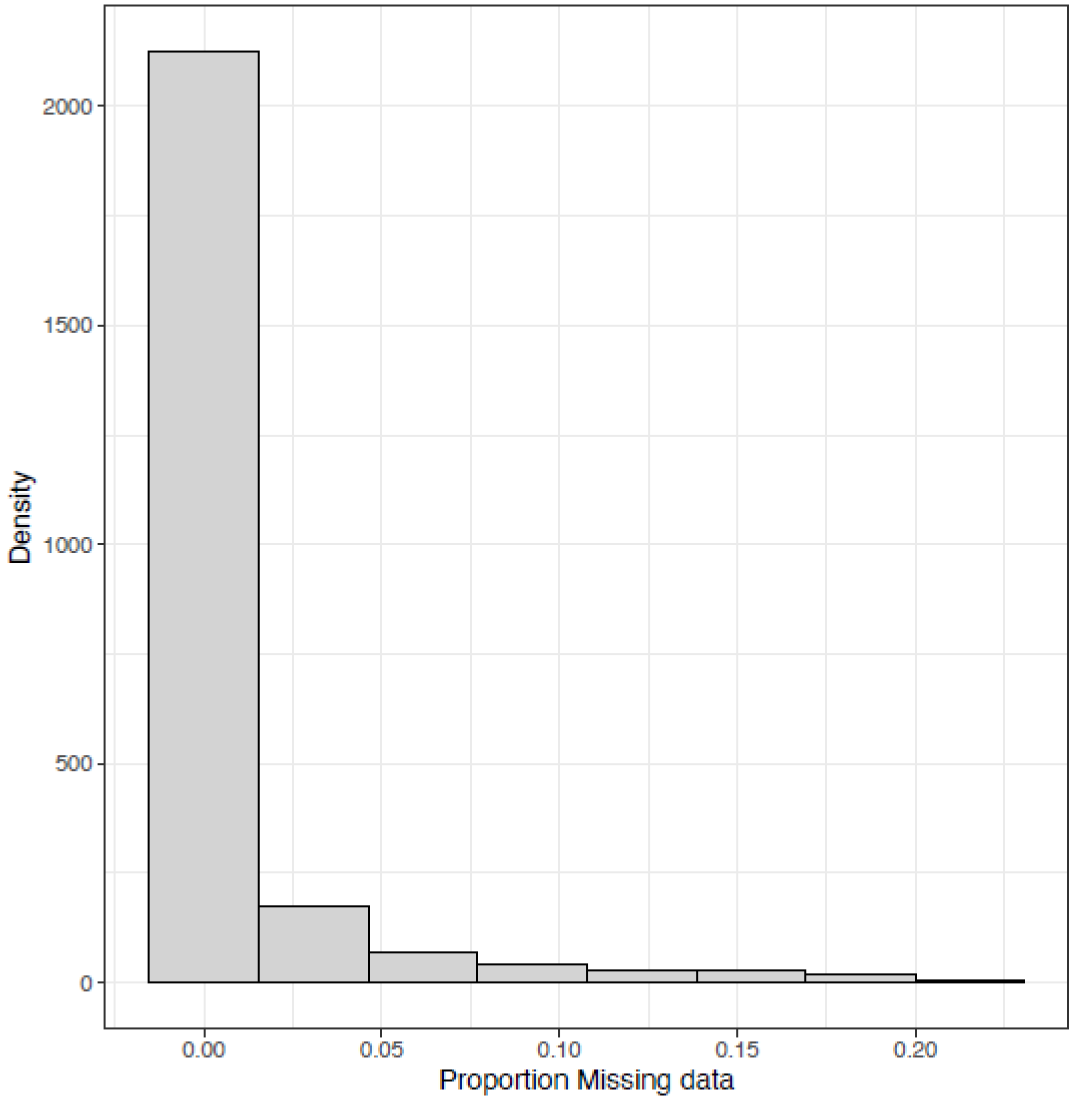

Supplementary Figure S3. Proportion of missing data (SNP markers) distribution in the AYB population

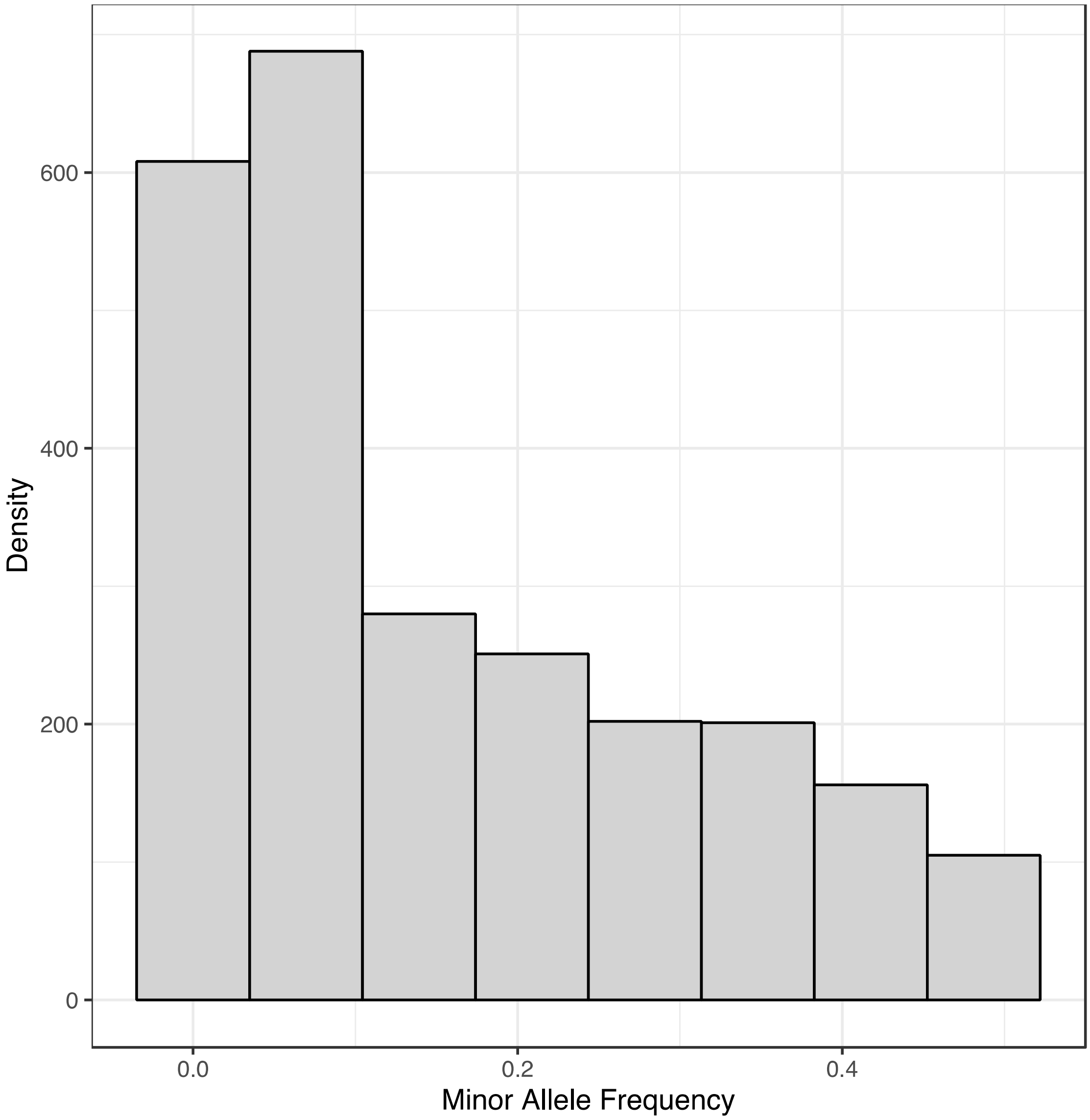

Supplementary Figure S4. Minor allele frequency distribution in the AYB population

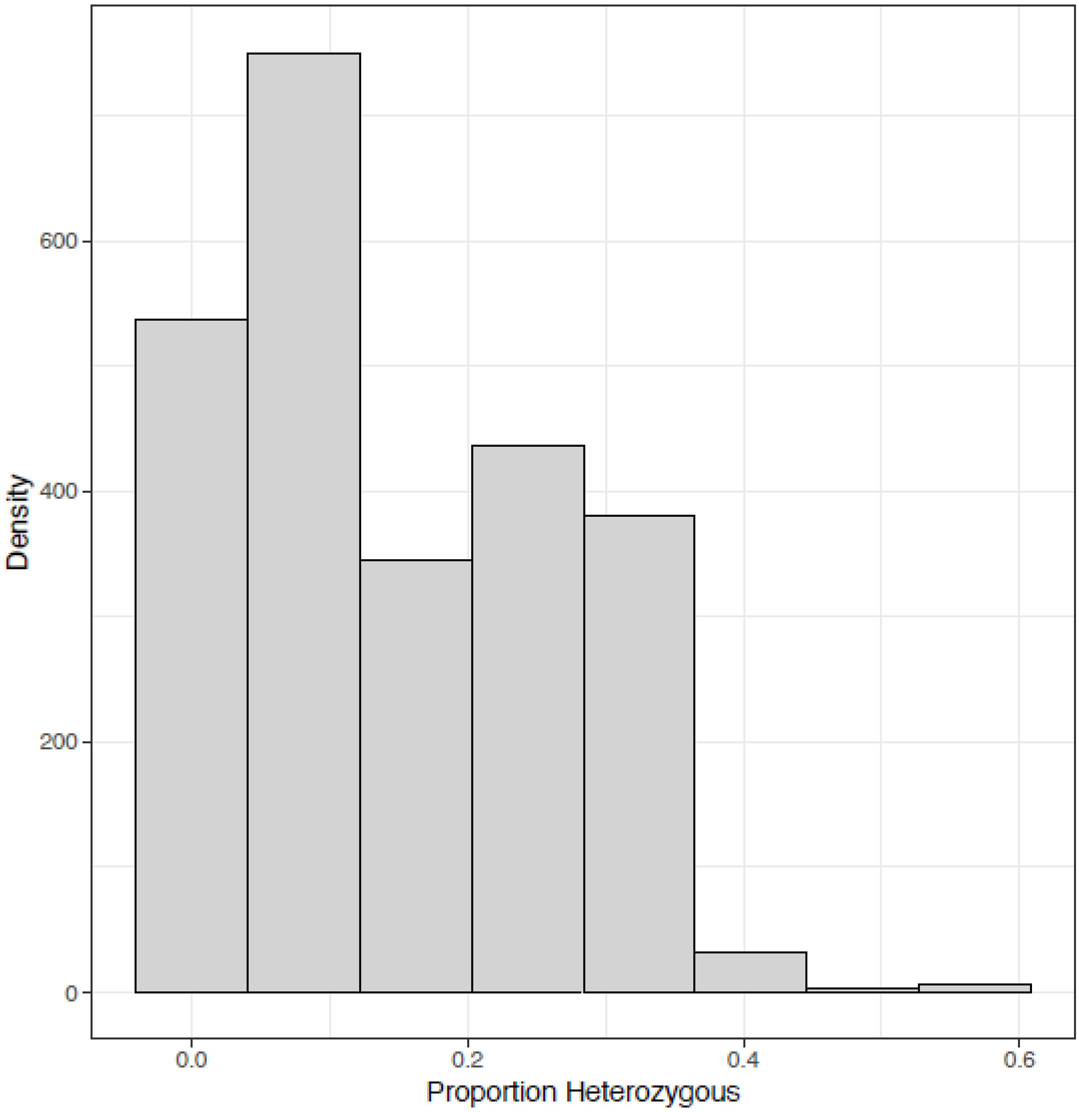

Supplementary Figure S5. Proportion of heterozygous (SNP markers) distribution in the AYB population

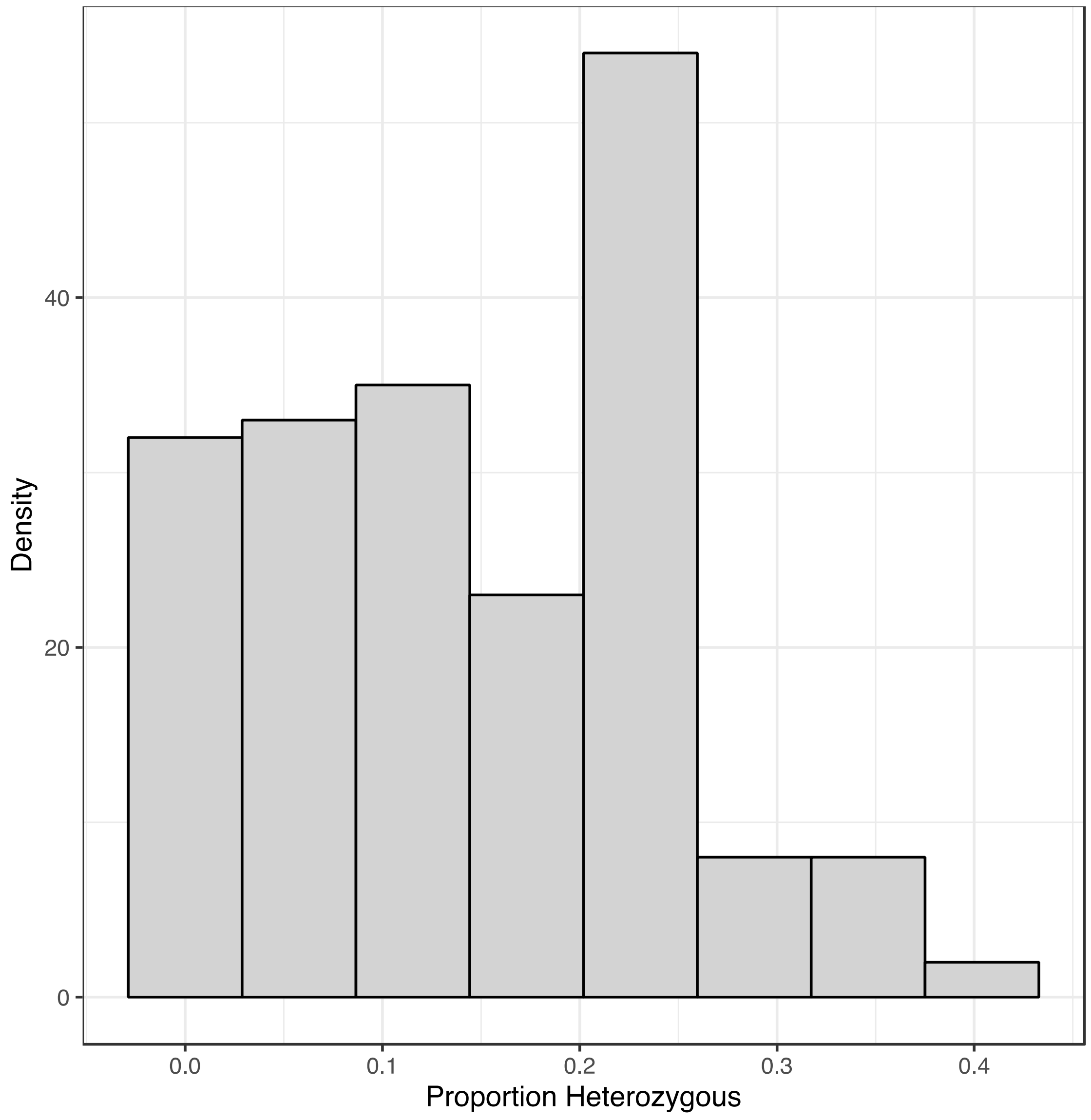

Supplementary Figure S6. Proportion of heterozygous (based on genotypes) distribution in the AYB population

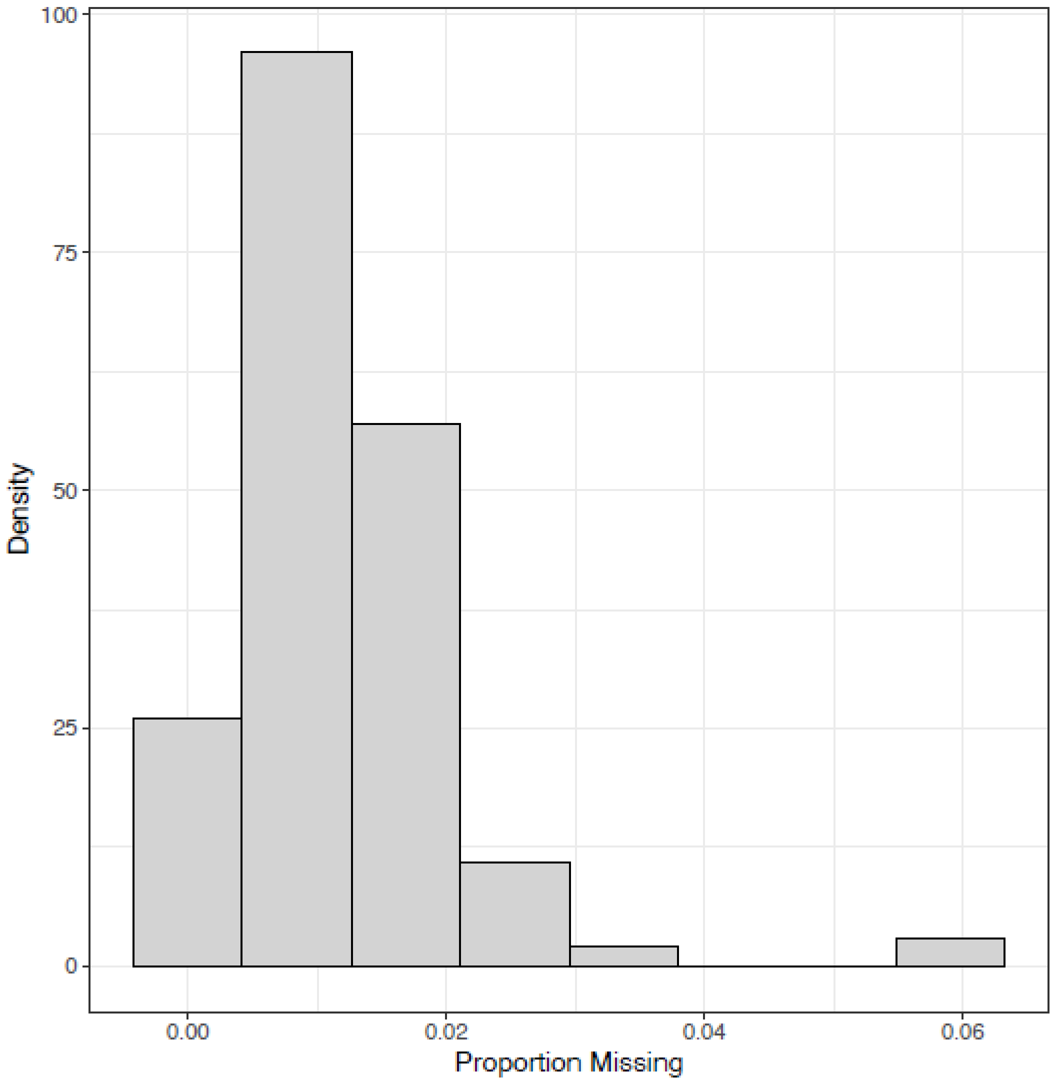

Supplementary Figure S7. Proportion of missing data (based on genotypes) distribution in the AYB population

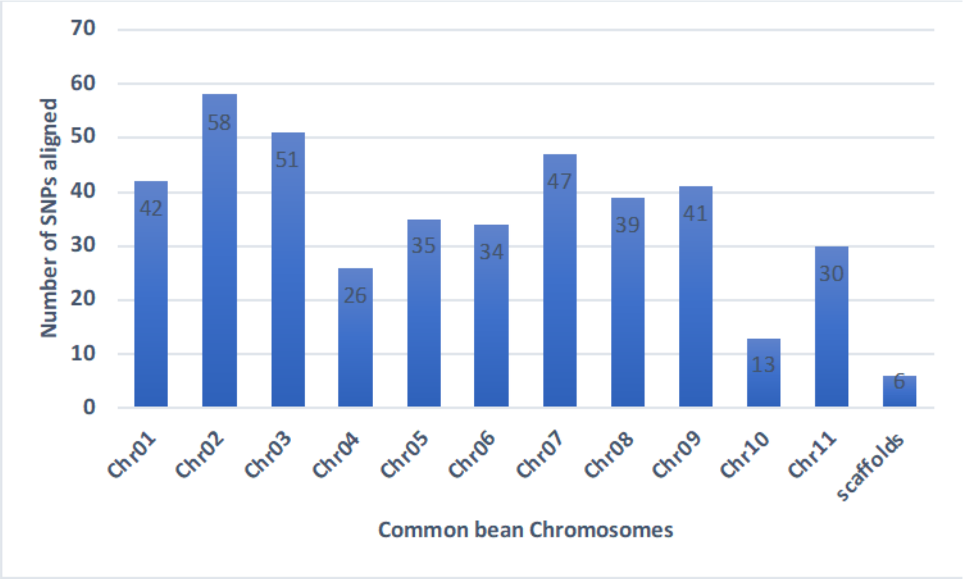

Supplementary Figure S8. African yam bean SNPs aligned on different chromosomes of common bean genome
